# Autophagy-related molecular clusters identified as indicators for distinguishing active and latent TB infection in pediatric patients

**DOI:** 10.1186/s12887-024-04881-1

**Published:** 2024-06-19

**Authors:** Yang Yu, Jie Hua, Liang Chen

**Affiliations:** 1 Department of Pediatric, Nanjing Lishui People’s Hospital, Zhongda Hospital Lishui Branch, Southeast University, Nanjing, China; 2 Department of Gastroenterology, The First Affiliated Hospital of Nanjing Medical University, Nanjing, China; 3 Department of Infectious Diseases, Taikang Xianlin Drum Tower Hospital, Affiliated Hospital of Medical College of Nanjing University, Qixia District, NO 188, Lingshan North Road, Qixia District, Nanjing, 210046 China

**Keywords:** Autophagy, Molecular cluster, Model, Active tuberculosis, Latent tuberculosis, Children

## Abstract

**Background:**

Autophagy is crucial for controlling the manifestation of tuberculosis. This study intends to discover autophagy-related molecular clusters as biomarkers for discriminating between latent tuberculosis (LTBI) and active tuberculosis (ATB) in children through gene expression profile analysis.

**Methods:**

The expression of autophagy modulators was examined in pediatric patients with LTBI and ATB utilizing public datasets from the Gene Expression Omnibus (GEO) collection (GSE39939 and GSE39940).

**Results:**

In a training dataset (GSE39939), patients with LTBI and ATB exhibited the expression of autophagy-related genes connected with their active immune responses. Two molecular clusters associated with autophagy were identified. Compared to Cluster 1, Cluster 2 was distinguished through decreased adaptive cellular immune response and enhanced inflammatory activation, according to single-sample gene set enrichment analysis (ssGSEA). Per the study of gene set variation, Cluster 2’s differentially expressed genes (DEGs) played a role in synthesizing transfer RNA, DNA repair and recombination, and primary immunodeficiency. The peak variation efficiency, root mean square error, and area under the curve (AUC) (AUC = 0.950) were all lowered in random forest models. Finally, a seven-gene-dependent random forest profile was created utilizing the CD247, MAN1C1, FAM84B, HSZFP36, SLC16A10, DTX3, and SIRT4 genes, which performed well against the validation dataset GSE139940 (AUC = 0.888). The nomogram calibration and decision curves performed well in identifying ATB from LTBI.

**Conclusions:**

In summary, according to the present investigation, autophagy and the immunopathology of TB might be correlated. Furthermore, this investigation established a compelling prediction expression profile for measuring autophagy subtype development risks, which might be employed as possible biomarkers in children to differentiate ATB from LTBI.

**Supplementary Information:**

The online version contains supplementary material available at 10.1186/s12887-024-04881-1.

## Background

Tuberculosis (TB), caused by *Mycobacterium tuberculosis* (Mtb) infection, is one of the top ten causes of death worldwide [1]. The 2020 Global Tuberculosis Report estimates that 1.4 million people died from TB in 2019 and that there were over 10 million new cases [2]. One-third of the world’s population is thought to be Mtb-infected, and 5–15% of them are predicted to acquire TB at some time in their life. The risk is higher in young younger children [3]. Particularly in young individuals, managing TB can be a challenging, drawn-out process that typically leads to inadequate compliance from patients [4]. The parameters that eventually influence the transition between latent TB (LTBI) and active TB (ATB) infection are still not fully understood, and clinically differentiating between these two disease states is still difficult, even though the fact that accomplishing so is essential for providing adequate care and halting the rapid spread of TB. The tuberculin skin test (TST) and the interferon release assay (IGRA) are the two most often used procedures to diagnose tuberculosis (TB). However, neither can consistently distinguish ATB from LTBI [5].

The diagnostic procedures might also be inaccurate in individuals with co-morbid HIV infection or malnutrition, or TB. On the other hand, false-positive results may occur in cases with non-tuberculous mycobacterial infection or after Bacillus Calmette-Guérin (BCG) vaccination [6]. Therefore, developing novel diagnostic markers that can accurately distinguish between these two types of Mtb infection is crucial. Host cell apoptosis is essential for controlling Mtb infection [7, 8]. Numerous genetic fingerprints that indicate the pathogenic process and serve as unique biomarkers for separating ATB from LTBI have been discovered [7–9]. In the body’s regular physiological functioning, autophagy plays a role in maintaining cellular homeostasis and survival. Pathogens (protists/protozoa, fungi, bacteria, and viruses), damaged organelles, and damaged proteins that the proteasome cannot break down are all degraded via autophagy [10].

Mycobacteria entering alveolar macrophages are engulfed by phagosomes that fuse with lysosomes, leading to the destruction of the bacteria and thus preventing their replication in the cells [11]. IFN-γ production rises in response to Mtb infection, activating autophagy and Mtb transport to the lysosomes. IFN-γ is thus associated with protective immunity against TB and the induction of autophagy [12]. Furthermore, research has demonstrated the importance of several autophagy components, including ATG5, ATG12, ATG16L1, p62, NDP52, BECN1, and LC3, for controlling Mtb infection, which enhances autophagy to eliminate intracellular Mtb [13, 14].

Nevertheless, most of these studies were carried out in vitro, and there is a shortage of information regarding autophagy in Mtb-infected clinical samples, particularly in young individuals. Here, using a bioinformatics-based methodology and gene expression profiles, the usefulness of autophagy-associated molecular clusters as indicators for differentiating LTBI and ATB in children was examined.

## Methods

### Data source

Datasets were extracted from the Gene Expression Omnibus platform (http://www.ncbi.nlm.nih.gov/geo). Relevant information was obtained from research involving HIV-negative children under the age of 15 whose samples were gathered before initiating anti-mycobacterial treatment. Using these requirements, the 2 largest analytical datasets were determined. The microarray training dataset GSE39939 included whole-blood samples from LTBI (*n* = 14) and ATB (*n* = 52) Kenyan pediatric patients. This dataset was used to construct a prediction model based on the expression profiles of autophagy-related molecular clusters. The external validation dataset was the GSE39940 microarray, which contained whole-blood samples from 54 South African and 70 Malawian patients. The detailed clinical characteristics of the patients from the two cohorts are given in Supplementary file 1.

**Fig. 1:**
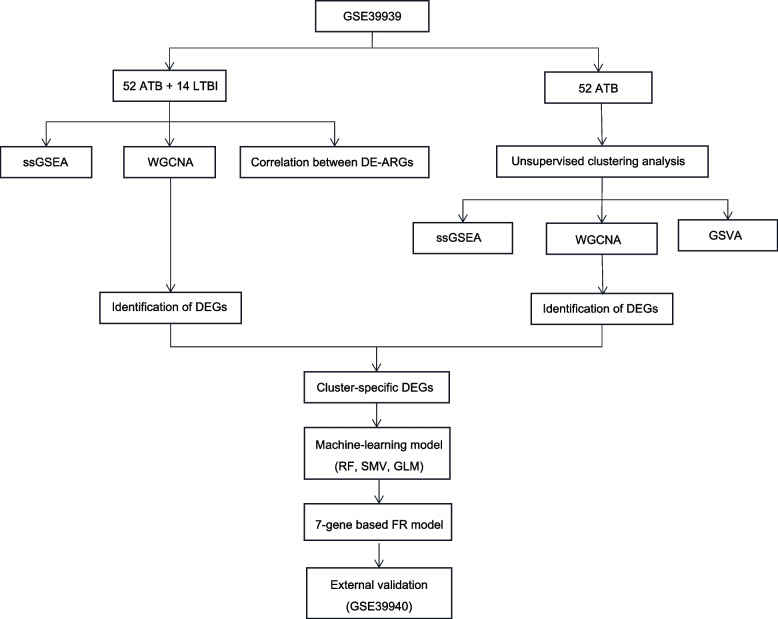
The study flow chart

The latter dataset was used for the validation of identified hub genes. Based on Mtb isolation and culture in respiratory samples, negative Mtb cultures in conjunction with clinical and radiological characteristics compatible with ATB or clinical symptoms consistent with TB, all patients with ATB were identified. On follow-up, LTBI was determined based on verified contact with persons with positive TB smear results and a positive IGRA or TST result but no clinical or radiological symptoms of ATB. The ComBat method was used to normalize the raw genomic expression profiles for GEO datasets (Fig. 1).

### Analysis of immune-cell infiltration (ICI) and autophagy-linked genomic expression in young LTBI and ATB patients

The expression profiles of 232 autophagy-related genes** (**ARGs) (Supplementary file 2) for ATB and LTBI in children were analyzed using the training dataset. In order to establish the relative ICI values in the training data set, the single-sample gene set enrichment analysis (ssGSEA) algorithm (v 1.58.0) was utilized (Supplementary file 3). Violin plots were used to analyze the differentiating expression of immune-infiltrating cells, and the R (v. 4.2.3) ‘ggplot2’ package (v 0.4.0) was employed to analyze the Spearman correlations between ICI and ARGs.

### Unsupervised clustering of TB pediatric patients

For unstructured clustering analysis and k-means classification of the 52 ATB data into different subtypes (1000 iterations), the ‘ConsensusClusterPlus’ package (v. 1.60.0) in R was used. This was accomplished on the basis of the expression profiles of differentially expressed ARGs (DE-ARGs). The optimal number of clusters was thoroughly found using the cumulative distribution function (CDF) curve, consistent cluster score (> 0.8), and consensus matrix with a peak subtype k-value of 9.

### Assessment of the Gene set variation analysis (GSVA)

The GSVA enrichment assessment was performed using the ‘GSVA’ R program (v. 1.44.5) to measure improved gene variants between ARG clusters. Using the ‘c2.cp.kegg. Symbols’ in the MSigDB database (https://ngdc.cncb.ac.cn/databasecommons/database), additional analysis was carried out. In order to assess the relationships between the various ARG clusters and biological pathways, the ‘limma’ R program (v. 3.52.4) was utilized, and the | GSVA score t-value |> 2 and p-value < 0.05 conferred statistically significant value.

### Evaluation of weighted gene co-expression network analysis (WGCNA)

WGCNA was executed in R using the “WGCNA” package (v.1.72–1) to find the co-expression modules. A second WGCNA analysis utilizing the best 25% of highly diverse genes was carried out to verify the validity of the results. A weighted adjacency matrix was established, and an ideal soft power was selected to transform it into a topological overlap matrix (TOM). Using a hierarchical clustering tree approach with a 100-module minimum, modules were collected using the TOM dissimilarity measure (1-TOM). In order to represent the global expression profiles of the genes within specific modules, random colors were utilized for both module eigengenes (MEs) and individual modules. Modular significance (MS) served as a visual representation of the relationship between disease status and modules. Gene significance (GS) is the genetic relationship between a gene and a clinical characteristic.

### Construction of a predictive model through several machine learning techniques

Machine learning models (MLM) consisting of support vector machine (SVM), random forest (RF), and generalized linear models (GLM) were used to evaluate the ARG clusters using the R “caret” package (v. 6.0–94). Differentially expressed genes (DEGs) particular to a specific cluster have been selected as the explanatory variables and response. Patients with tuberculosis from the GSE39939 dataset were randomly assigned into a testing set (30%) and a training set (70%). Grid search utilizing the “caret” software optimizes model parameters automatically. The fivefold cross-validation method was used to assess each model after it had been developed with standard variables. The three models were described, and their significance for the residual distribution and features was visualized using the “DALEX” package (v.2.4.2). The ‘pROC’ R program (v. 1.18.0) was employed to calculate the areas under the receiver operating characteristic (ROC) curves (AUCs). The seven top-ranking genes were regarded as the dominant ATB-predicting genes, and the ideal MLMs were then identified. Finally, ROC curves were employed to confirm the diagnostic model’s precision.

### Development and verification of the nomogram model

The “rms” R package (v. 6.8–0) was used to generate a nomogram. Every indicator included an additional score; the “total score” was the total of all the predictor scorings listed above. Employing decision curve analysis (DCAB), it became feasible to measure the nomogram’s predictive values. The external validation dataset (GSE39940) was used in ROC studies. The ‘pROC’ R package was utilized for analyzing ROC curves to confirm the prediction model’s ability to differentiate between patients with LTBI and ATB. A P < 0.05 was deemed as a statistically significant value.

## Results

### Autophagy modulator dysregulation and immune response activation in ATB and LTBI pediatric patients

**Fig. 2:**
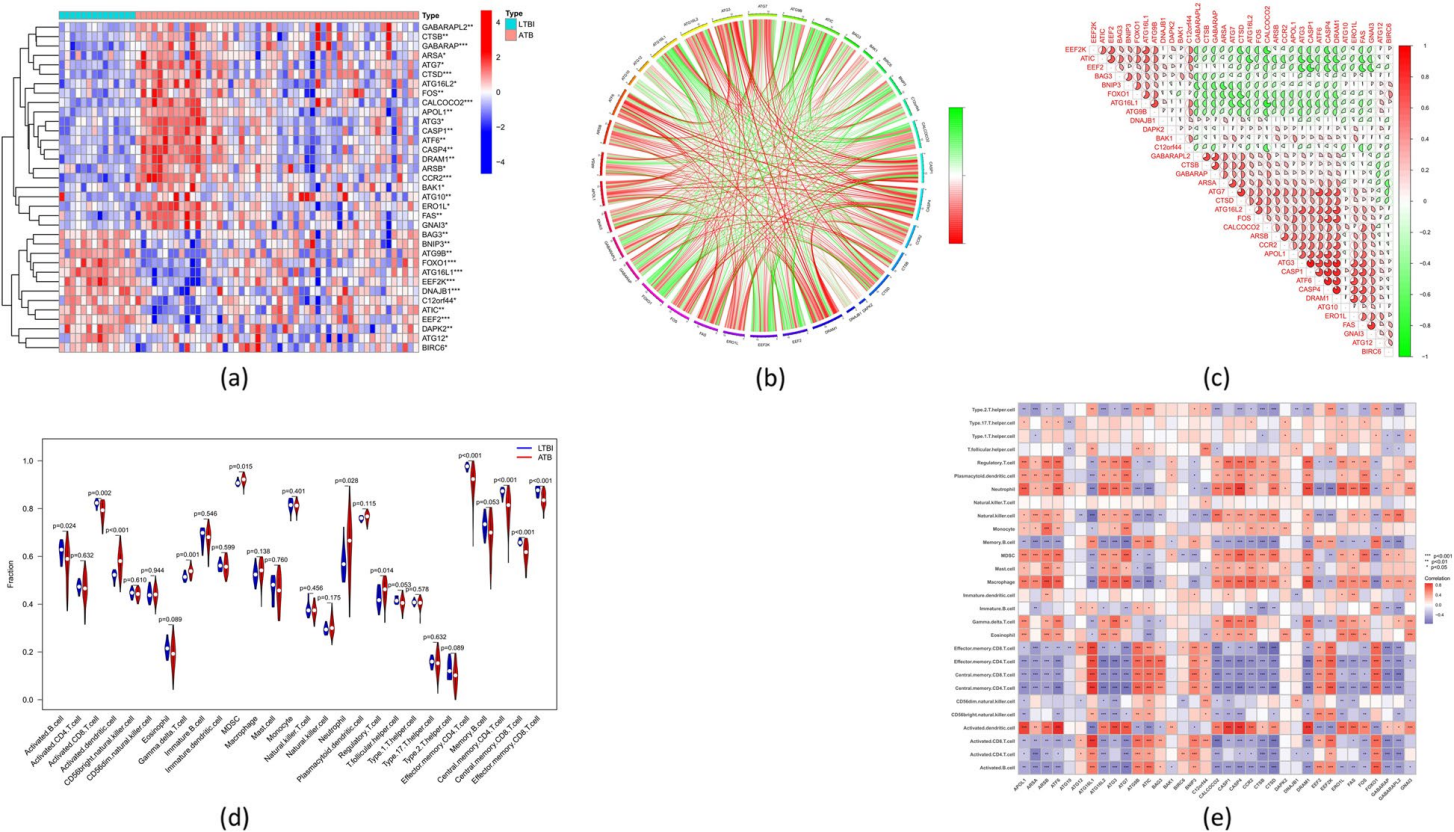
Identification of dysregulated ARGs in ATB pediatric patients. **a** Heatmap illustrating the expression patterns of 35 differentially expressed ARGs (DE-ARGs) across patient samples, with red indicating positive correlation, green indicating negative correlation, and white indicating no correlation. **b** The circleplot showing the correlations of these genes. The red and green colors of the scale represent positive and negative correlations, respectively. **c** Correlation analysis of 35 DE-ARGs. The red and green colors of the scale represent positive and negative correlations, respectively. **d** Comparison of the relative abundances of infiltrated immune cells between ATB and LTBI patients. *p < 0.05, **p < 0.01, ***p < 0.001

A total of 35 DE-ARGs were identified among the pediatric patients with LTBI and ATB (Fig. 2a). The associations between these DE-ARGs are shown in the gene-relationship network diagram (Fig. 2b). Several of these DE-ARGs had positive associations (ATG9B with EEF2K; ATIC1 and EEF2), while others had negative associations (ATG16L2 with EEF2K; BAG3 and ATG16L1) (Fig. 2c).

The training dataset was evaluated using the ssGSEA algorithm, which revealed prevention of the adaptive cellular immune response in ATB patients compared to LTBI patients, as evidenced by reduced penetration of activated CD8 cells and effector memory CD8 T-cells and central and an increased inflammatory response (including noticeably increased monocyte and neutrophil infiltration) (Fig. 2d). Correlation analysis also revealed that the DE-ARGs were linked to immune cells, indicating that they are crucial in controlling immune cell infiltration during TB infection (Fig. 2e).

### Identification of autophagy-linked molecular clusters in children with TB

**Fig. 3:**
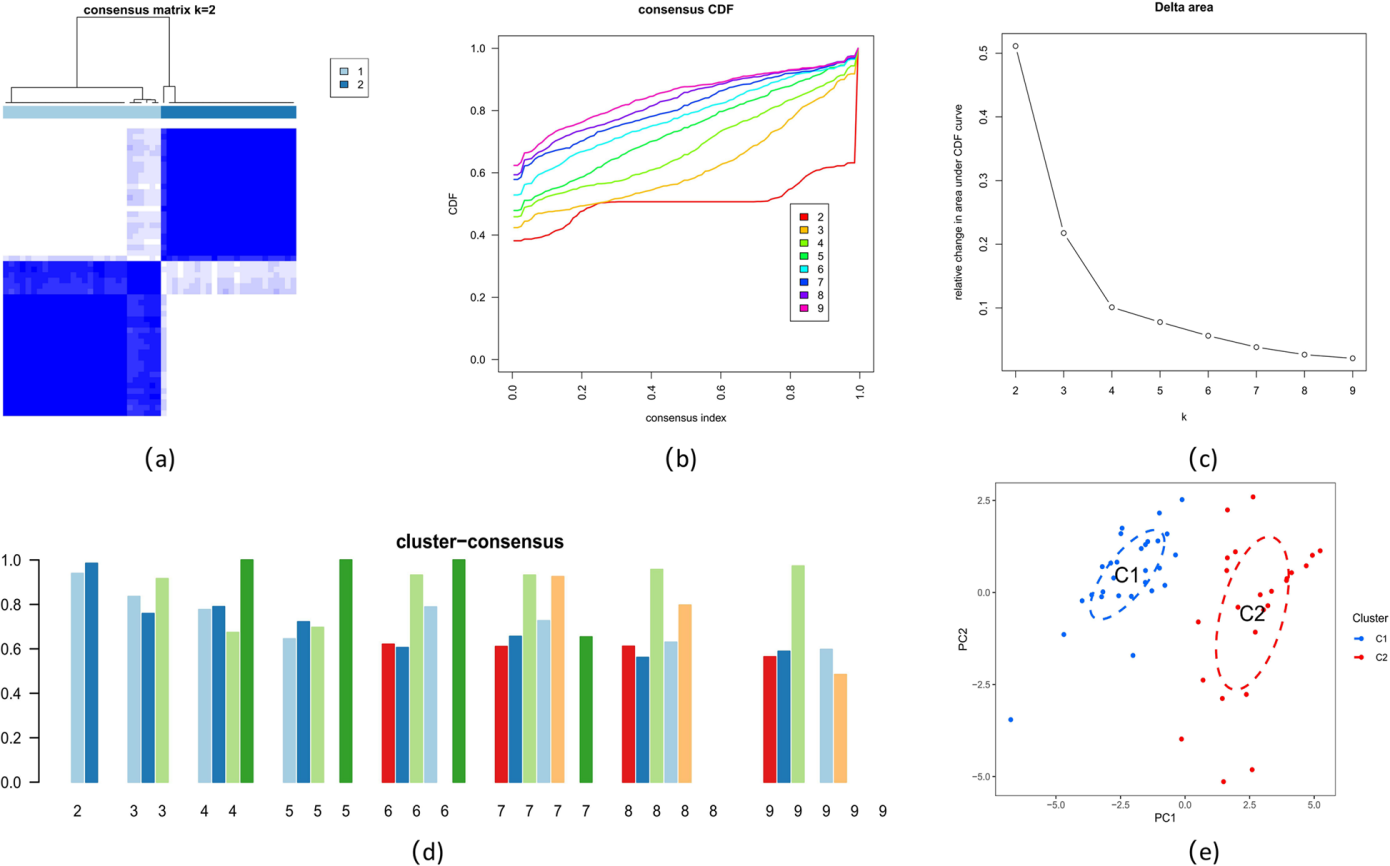
Identification of autophagy-related molecular clusters in ATB. **a** Consensus clustering matrix when k = 2. **b** CDF delta area curves. **c** The score of consensus clustering. **d** Heatmap representation of the non-negative matrix factorization output. **e** t-SNE plot visualizing the distribution of two subtypes

The expression profiles of the 35 DE-ARGs were categorized using a consensus clustering technique. With k-values = 2, the most stable clusters were observed. A minimized range with cumulative distribution function (CDF) curves that differed between a consensus index of 0.2 and 0.8 (Fig. 3a-b). Areas under CDF curves (k; k-1) where k = 2 to 9 revealed any variance across both CDF curves (Fig. 3c). The individual subtype’s consistency score at k = 2 was > 0.8 (Fig. 3d). The analysis of the two subtypes using t-Distributed Stochastic Neighbor Embedding found significant variation (Fig. 3e).

### Variations in autophagy modulators, immune-infiltration profiles, and functional annotation in autophagy clusters

**Fig. 4:**
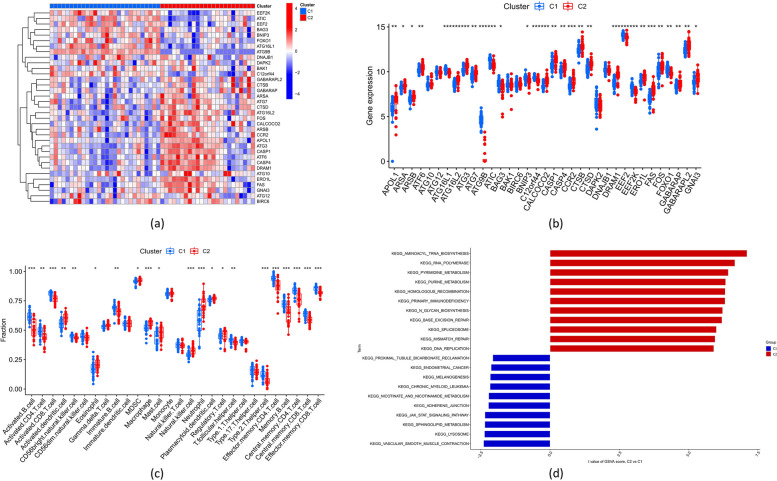
Identification of molecular and immune characteristics between the two autophagy-related molecular clusters. **a** Heatmap showing the differential expression patterns of 35 DE-ARGs between two distinct autophagy clusters. **b** Boxplots detailing the expression levels of each of the 35 DE-ARGs across the two autophagy clusters. **c** Boxplots depicting variations in immune cell infiltration between the two autophagy clusters. **d** Differences in hallmark pathway activities between Cluster 1 and Cluster 2 samples ranked by t-value of GSVA method. *p < 0.05, **p < 0.01, ***p < 0.001

Landscapes of expression for specific ARGs were evaluated between the two autophagy patterns. Several ARGs (such as ATIC, EEF2, BAG3, and FOXO1) were up-regulated in Cluster 1, while others (ATG7, CTSD, ATG16L2, and CALCOCO2) were up-regulated in Cluster 2 (Fig. 4a-b). Analysis of immune infiltration indicated increased access to neutrophils, dendritic cells, and macrophages in cluster 1. Still, it decreased the penetration of NK cells, active/memory CD4 T cells, and memory CD8 T cells (Fig. 4c).

Cluster 2 was primarily implicated in the transfer of RNA biogenesis, DNA repair and recombination, and primary immunodeficiency pathways, according to the GSVA analysis. In contrast, Cluster 1 genes were primarily involved in the pathways of inflammation response, chronic lymphocytic leukemia, lysosome, and metabolism of cofactors and lipids, among others (Fig. 4d).

### Gene modules and co-expression network

**Fig. 5:**
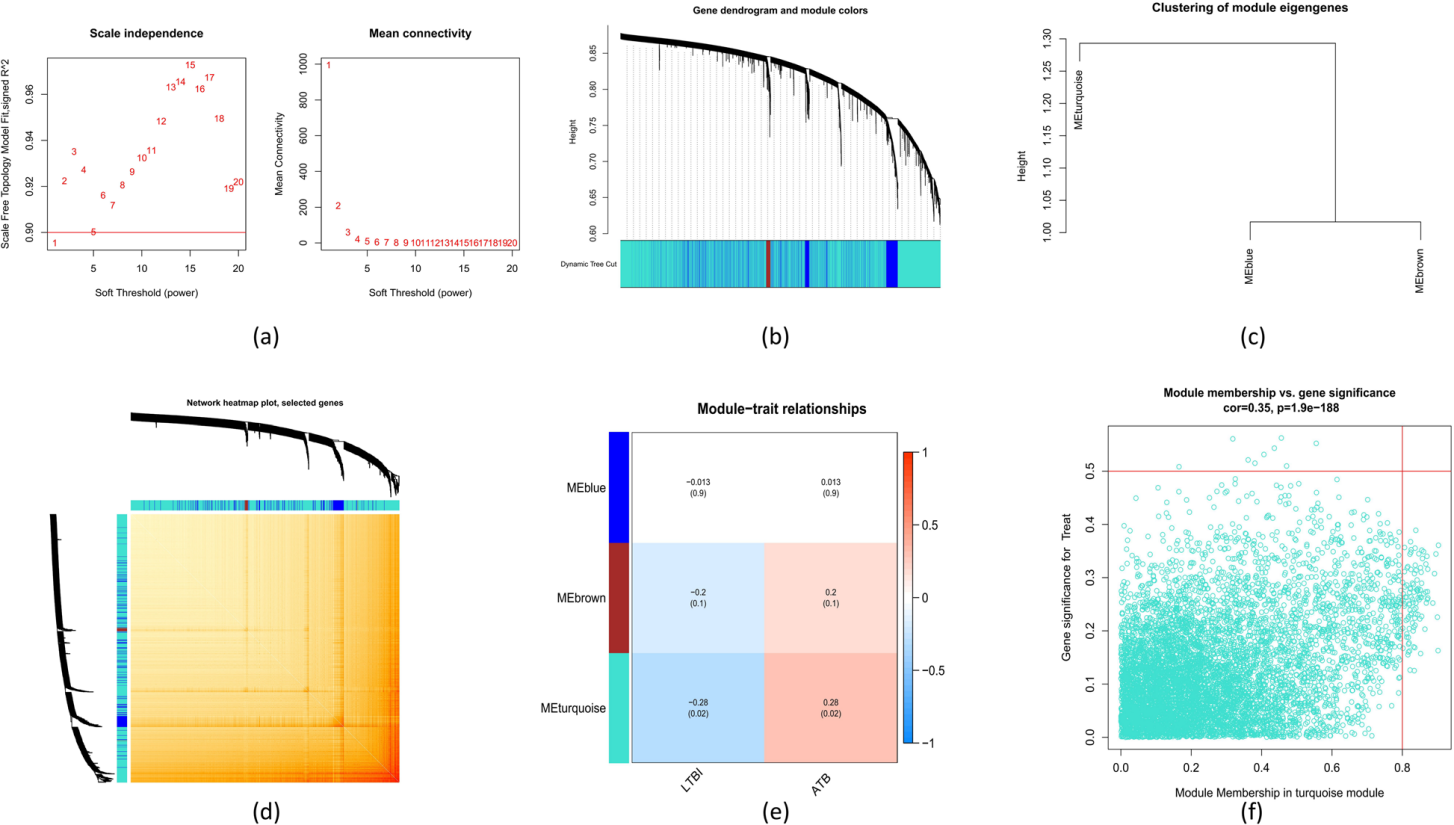
Co-expression network analysis of differentially expressed genes in ATB patients. **a** Determination of the optimal soft threshold power. **b** Cluster tree dendrogram showing co-expression modules; different colors denote distinct modules. **c** Visualization of clustering for module eigengenes. **d** Heatmap illustrating the correlations among three co-expression modules. **e** Correlation analysis between module eigengenes and various clinical statuses of ATB patients, with red and blue indicating positive and negative correlations, respectively. **f** Scatter plot showing the relationship between module membership within the turquoise module and gene significance for ATB

Using WGCNA, a co-expression network has been developed in the training set to identify the crucial modules related to ATB. Using a cut height = 0.25 and soft-thresholding power = 1 (scale-free *R*^2^ = 0.9), the implication of the hub genes was measured. As a result, three modules were selected. The relationship between sample attributes and ME values served as the measurement and heat mapping basis (Fig. 5a-d). The figure demonstrates that the clinical state was strongly correlated with the turquoise module (cor = 0.28, *p* = 0.02) (Fig. 5e). Additionally, it was discovered that the blue module and genes connected with it have a positive correlation (cor = 0.35, *p* = 1.9 e-188) (Fig. 5f).

**Fig. 6:**
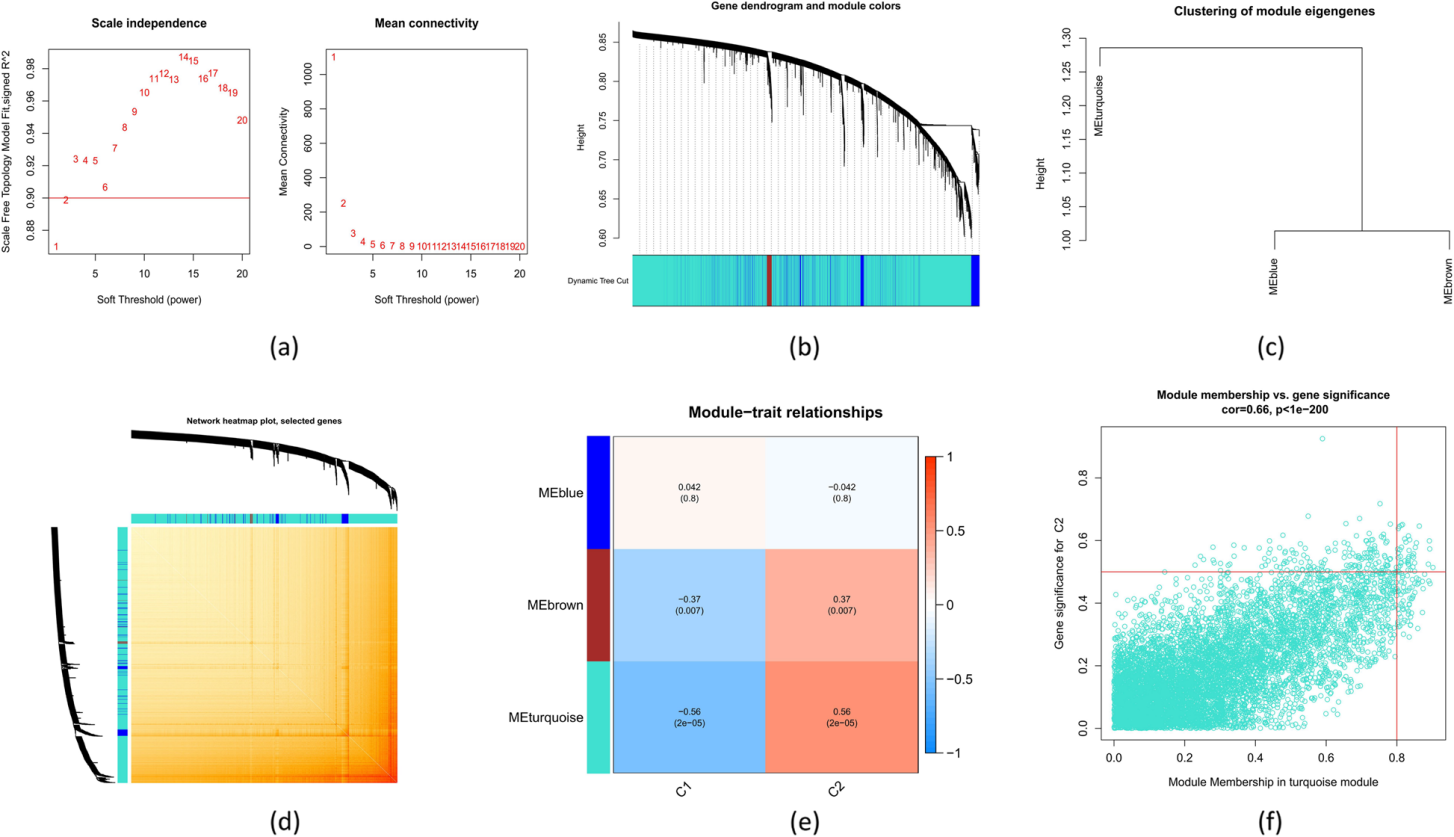
Co-expression network of differentially expressed genes in the two autophagy-related molecular clusters. **a** Cluster tree dendrogram illustrating distinct co-expression modules, with each color representing a different module. **c** Clustering of module eigengenes to show relationships between modules. **d** Heatmap displaying the correlation among the three co-expression modules. **e** Correlation heatmap between module eigengenes and the two autophagy-related molecular clusters, with rows representing modules and columns representing clusters. The red and blue colors of the scale represent positive and negative correlations, respectively. **f** Scatter plot between module membership in the turquoise module and the gene significance for Cluster 2

Hub-gene modules that were closely linked with autophagy-related molecular clusters were also analyzed through WGCNA. The *R*^2^ = 0.9 and β = 1 reflected optimal soft-threshold variables for constructing scale-independent networking (Fig. 6a). The TOM for all the genes is displayed as a heatmap, and three modules were particularly important (Fig. 6b-d). The relationship evaluation of clinical variables associated with the module (Cluster 1 and Cluster 2) demonstrated a significant relationship in the ATB subtypes and blue module (cor = 0.56, *p* = 2e-5) (Fig. 6e), with a positive relationship between the module-associated genes and turquoise module (cor = 0.66, *p* < 1e-200, Fig. 6f).

### Identification of autophagy-related molecular cluster-specific DEGs

**Fig. 7:**
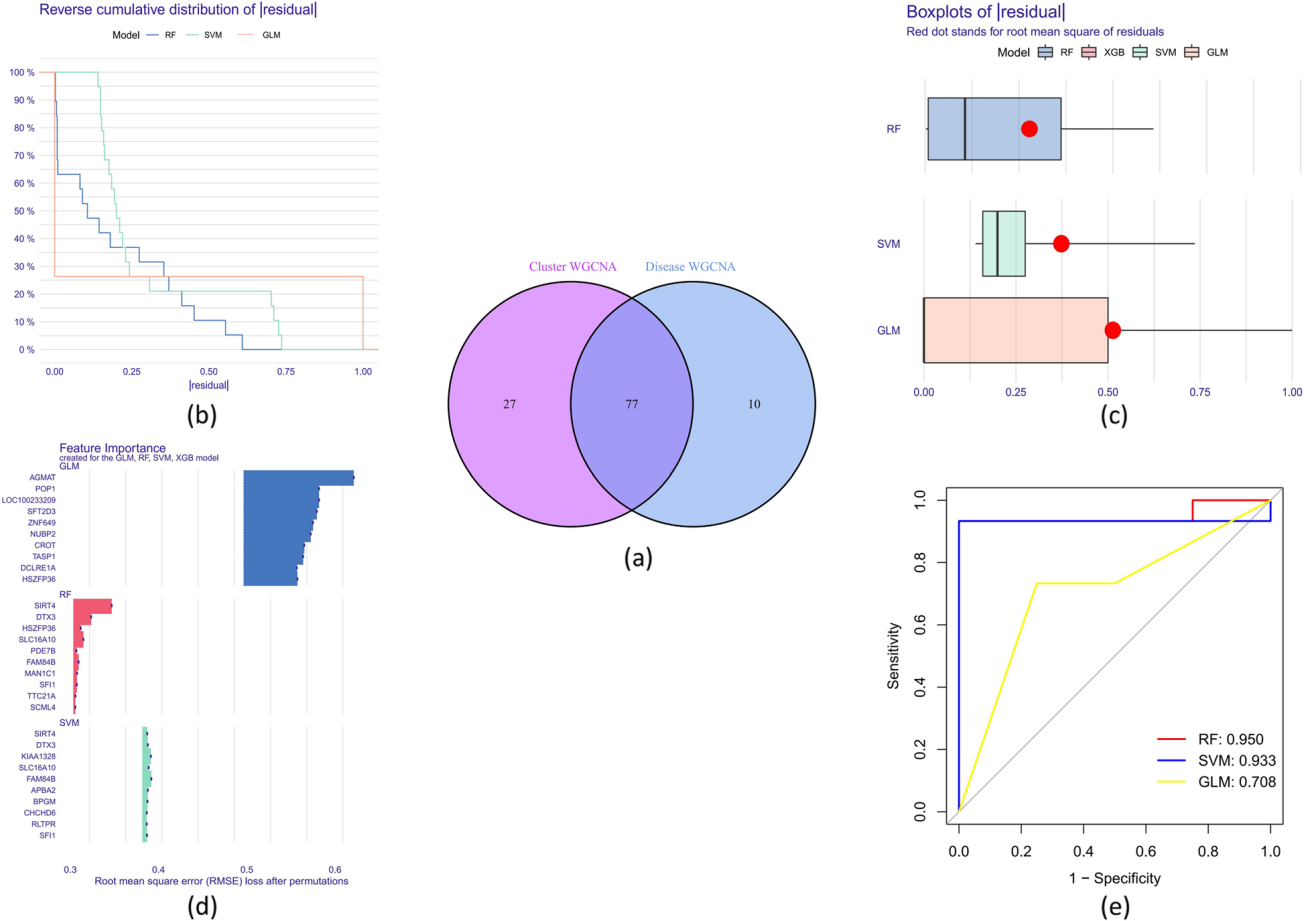
Identification of cluster-specific DEGs and construction of machine models. **a** Venn diagram showing the intersections of module-related genes from autophagy-related clusters with those identified in the training dataset. **b** Cumulative residual distribution of each machine learning model. **c** Boxplots visualizing the residuals of each machine learning model used in the study; the red dot within each boxplot indicates the root mean square error (RMSE). **d** Important features in RF, SVM, and GLM machine models. **e** ROC analysis of four machine learning models in the training dataset

The assessment of relationships between module-associated genes in autophagy clusters and module-related genes in ATB and LTBI patients yielded 77 cluster-specific DEGs (Supplementary file 4 and Fig. 7a).

### Development and evaluation of machine learning models

RF, SVM, and GLM models were developed using the expression profiles of the 77 cluster-specific DEGs in the training set to discover cluster-specific genes with high diagnostic potential. The residual-earning model for the RF machine was significantly lower (Fig. 7b-c). Each model’s top 10 significant feature variables were consequently ranked on the basis of their root mean square error (RMSE) (Fig. 7d). Additionally, ROC curves were constructed (five-fold cross-validation-based) to evaluate the discerning activity of the three machine learning algorithms within the training set. In comparison to GLM (AUC = 0.707) and SVM (AUC = 0.933), the RF model’s area AUC peaked at 0.950 (Fig. 7e).

**Fig. 8:**
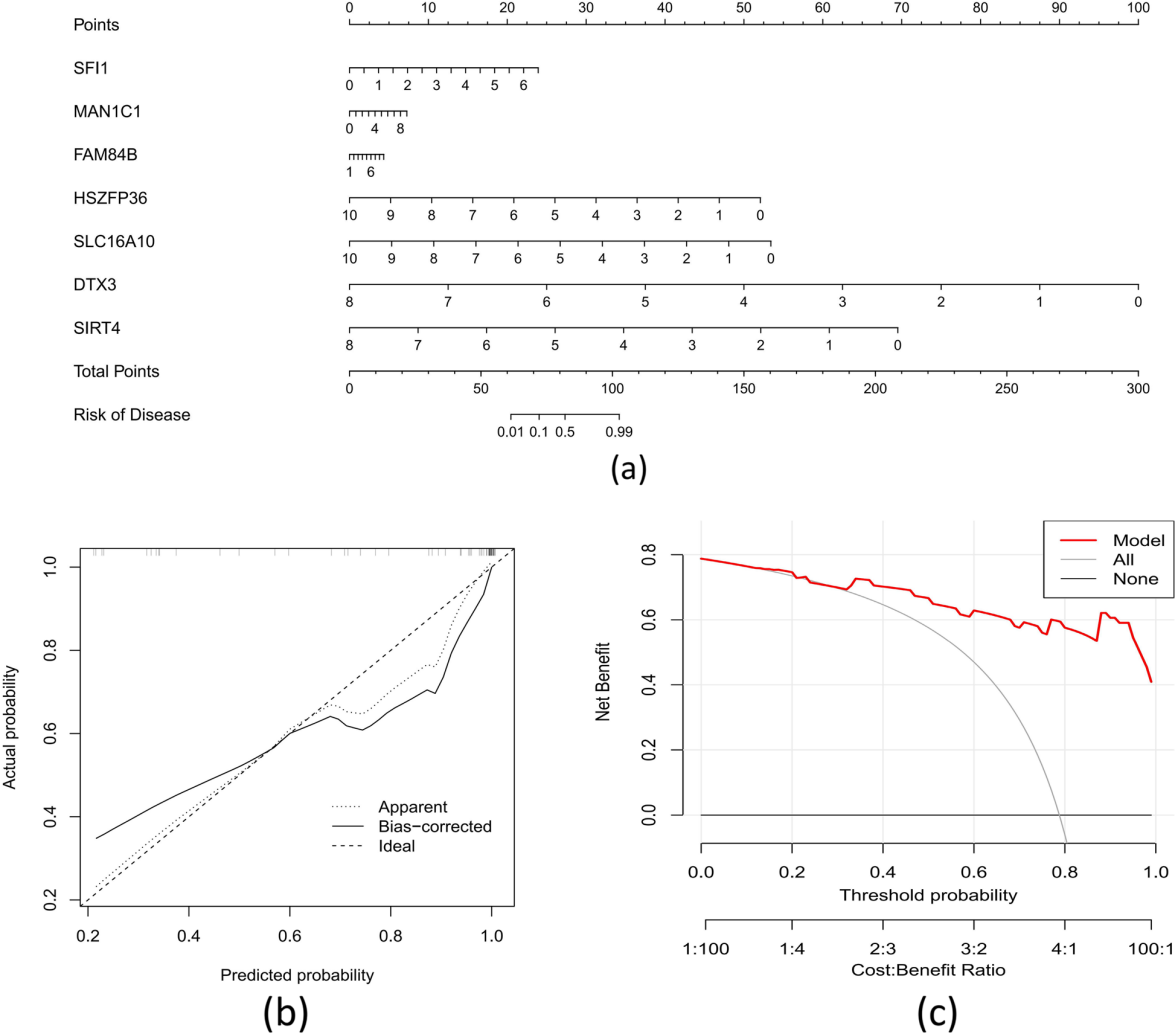
Construction of a nomogram model. **a** Construction of a nomogram for predicting the risk of ATB clusters based on the 5-gene-based FR model. Construction of calibration curve (**b**) and DCA (**c**) for assessing the predictive efficiency of the nomogram model

**Fig. 9:**
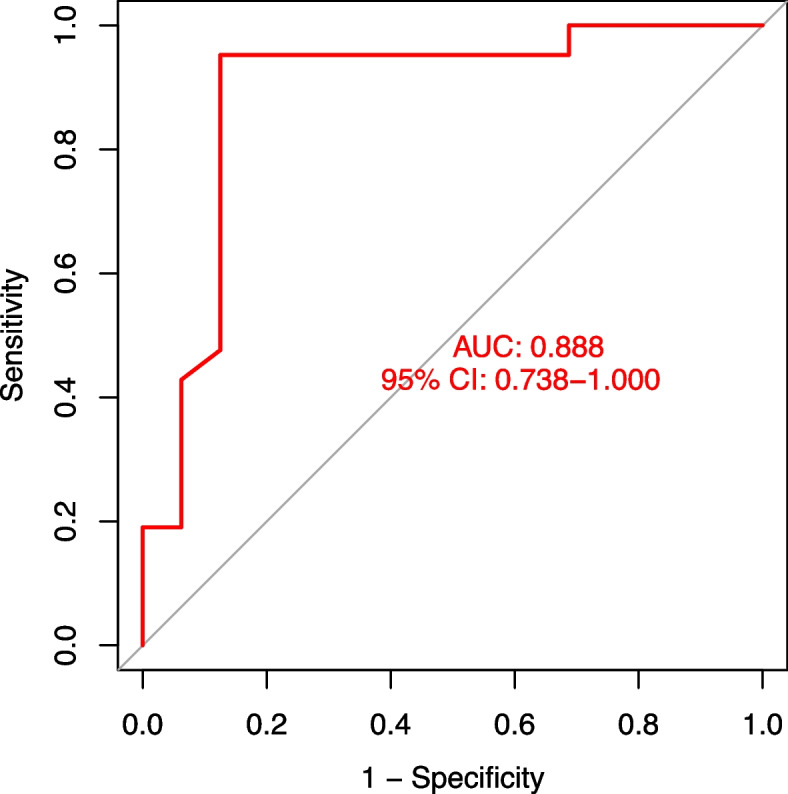
ROC analysis of the 7-gene-based FR model based on fivefold cross-validation in the external validation dataset (GSE39940)

The prediction performance of the RF model was evaluated further by developing a nomogram for determining the risk values of autophagy clusters in 52 ATB individuals from the training dataset (Fig. 8a). Based on the calibration curve, errors across actual/predicted ATB cluster risk were reduced (Fig. 8b), while DCA indicated this nomogram to have good accuracy (Fig. 8c). The 7-gene prediction model was then verified against an external validation dataset (GSE39940), with ROC curves indicating a 0.888 AUC value (Fig. 9).

## Discussion

Despite recent advances in treatment and diagnosis, tuberculosis remains a leading infectious disease causing significant comorbidity and mortality globally [1–5]. Current transcriptome investigations have identified several genes and gene expression patterns associated with tuberculosis pathogenesis. The death of host cells is critical for Mtb infection control, as it prevents mycobacterial growth and dissemination [7–9]. Apoptotic cell death, in particular, is a defense technique cells use to resist intracellular infections [8]. In contrast, necrotic death is unregulated, allowing Mtb to disseminate to proximal cells upon lytic death [9]. Autophagy is also crucial in the regulation of Mtb. However, the pathogen can prevent autophagosome acidification and subsequent lysosomal fusion, allowing it to proliferate and survive within infected macrophages. *In-vitro* investigations have shown that autophagy induced by rapamycin or starvation can reduce the lifespan of intracellular Mtb [10–14].

This investigation comprehensively analyzed DE-ARGs and immunological profiles between children with ATB and LTBI. ATB patients were classified into two subgroups according to their DE-ARG expression profiles, followed by an assessment of the biological functions of the cluster-specific genes. In order to determine ATB patients with distinct molecular clusters, an algorithm for prediction was developed using comparative analyses of various machine-learning algorithms. The model’s effectiveness was then validated against an external dataset, calibration curves, DCA, and a nomogram.

Increasing data suggest that the interaction of host immune responses and cell death pathways is critical for the development of ATB after LTBI [15, 16]. Consistent with previous studies [15–17], our study discovered that when compared to LTBI, the immune status of ATB individuals was categorized by lymphocyte suppression, demonstrated by substantial decreases in the B cell, CD8 + T cell, and CD4 + T cell populations, as well as activation of myeloid and inflammatory cells (monocytes, neutrophils, and macrophages). Through altering the development of granulomas, B cells and the associated antibodies they produce may influence the course of a Mtb infection [15]. Consequently, an unrestrained Mtb infection and the change from LTBI to ATB have been associated with a decreased lymphocyte response. Patients with ATB experience elevated inflammatory reactions that involve dendritic cells, monocytes, macrophages, and neutrophils in response to widespread bacterial presence and associated tissue damage [17].

Furthermore, this study found that several DE-ARGs (GABARAPL2, GABARAP, and CALCOCO2, etc.) were up-regulated in pediatric ATB patients. These genes have been identified to have a negative correlation with lymphocytes and a positive correlation with myeloid and inflammatory cells. Several DE-ARGs were downregulated (FOXO1, ATG9B, EEF2K, etc.), adversely correlated with inflammatory cells, and positively associated with lymphocytes. The dysregulation of ARGs and their relationship with immune cell populations underscore the potentially crucial role that autophagy can play in the immunopathogenesis of LTBI in children, eventually progressing to ATB. Using the expression profiles of 35 DE-ARGs, independent cluster analysis indicated the mechanisms regulating autophagy in juvenile ATB patients and identified two distinct autophagy-related molecular clusters. An ICI analysis revealed that Cluster 2 was characterized by the mobilization of myeloid and inflammatory cells and the inhibition of lymphocytes. Cluster 2 was predominantly enriched in transfer RNA biogenesis, DNA repair and recombination, and primary immunodeficiency, according to GSVA, based upon cluster-specific DEGs. These data revealed a stronger link between Cluster 2 and pediatric ATB immunopathology.

For the past twenty years, effective and precise bioinformatics techniques have been created to analyze expanding biological data for discoveries [18–20]. Machine-learning models have proven beneficial for combining data from various sources, enabling investigation of the interactions between demographic, environmental, and genetic variables in the emergence of multiple diseases. Therefore, the conclusions of these multifactorial investigations are more accurate and reliable than those of univariate analysis.

This study established a prediction model for pediatric ATB utilizing three machine-learning classifiers (RF, GLM, and SVM) based on the expression patterns of cluster-specific DE-ARGs. For classification or regression prediction, RF employs a variety of decision trees [18]. Previous researchers can construct a hyperplane with a maximum margin using the SVM technique to distinguish between negative and positive cases [19]. GLM was established as an expansion of multiple linear regression models to examine the association between normally distributed profiles and continuous/categorical independent variables [20]. Finally, it was demonstrated that the best AUC for predicting autophagy clusters in ATB patients was achieved via RF-based machine learning in this study.

As a result, the seven most essential genes (CD247, MAN1C1, FAM84B, HSZFP36, SLC16A10, DTX3, and SIRT4) were chosen to create an RF prediction model. An earlier investigation revealed that -mannosidase I (MAN1C1) contributes to cellular immunity during several chronic diseases, including infection with the hepatitis B virus [21]. Pathogens evade immune identification via DC-SIGN when a-mannosidase I expression increases, preventing the clearance of the etiologies and an efficient immune response [22]. The mitochondrial sirtulin (SIRT) family member SIRT4 performs deacetylase, substrate-specific deacetylase, lipoamidase, and ADP-ribosyltransferase activities [23]. Additionally, SIRT4 has been associated with cellular immunity to microorganisms [24]. SIRT4 overexpression in LPS-treated cells was discovered to increase steroidogenesis and decrease apoptosis, which facilitates the controlling of LTBI activation and Mtb infection [25].

Previous studies found that SIRT4 inhibits anti-inflammatory function, which supports inflammatory responses in ATB [26]. SLC16A10 encodes TAT1 or MCT10, an aromatic amino acid transporter [27]. During the early phase of Mtb infection in murine macrophages, SLC16A10 was significantly up-regulated and responsible for regulating host cell cholesterol efflux [28]. Mtb was discovered to promote cholesterol accumulation in cell walls, altering cell wall permeability and reducing rifampicin absorption, consequently exacerbating Mtb infection [29]. CD3zeta chain (CD3) is the term for CD247 [30]. CD247 is involved in T cell receptor phagocytosis and signal transduction (TCR) [31]. Moreover, chronic inflammation has been linked to the downregulation of the CD3zeta chain [32]. However, links between FAM84B, HSZFP36, and DTX3 and tuberculosis remain uncharacterized.

Our present research has some limitations. Such as, despite several attempts to explore all publicly accessible datasets, the study population size for this study was reasonably small, which may have compromised the validity of our outcomes. The current research found an association between ARGs and immune cells. However, it should be considered statistical rather than causative. Perhaps these host characteristics are exclusive to Mtb infection is still unknown. In addition, further comprehensive clinical data are necessary for verifying the prediction model’s performance level. Subsequently, microarrays have significant drawbacks (they do not analyze the entire genome, have high background signal levels, cannot detect alternative splicing, and are not quantitative). Moreover, in vitro and in vivo studies evaluating the action of these ARGs and related-molecular clusters are required to establish the processes underlying the pathophysiology of young Mtb infection.

In summary, our work thoroughly assessed the level of immune cell infiltration and gene expression associated with autophagy in LTBI and ATB patients. A seven-gene-based model was developed to predict autophagy clusters linked to ATB risk in children. The results of this research imply that autophagy is associated with the immunopathology of Mtb infection activation and show the potential for autophagy as a novel and accurate biomarker for distinguishing ATB from LTBI in young individuals.

### Supplementary Information

Supplementary Material 1.

Supplementary Material 2.

Supplementary Material 3.

Supplementary Material 4.

## Data Availability

Publicly available datasets were analyzed in this study. These data can be found in GSE37250 (https://www.ncbi.nlm.nih.gov/geo/query/acc.cgi?acc=GSE37250), GSE19491 (https://www.ncbi.nlm.nih.gov/geo/query/acc.cgi?acc=GSE19491), GSE28623 (https://www.ncbi.nlm.nih.gov/geo/query/acc.cgi?acc=GSE28623).

## Abbreviations

ATB: Active tuberculosis
LTBI: Latent tuberculosis infection
Mtb: *Mycobacterium tuberculosis*
ARGs: Autophagy-related genes
DEGs: Differentially expressed genes
ssGSEA: Single-sample gene set enrichment analysis
GSVA: Gene set variation analysis
WGCNA: Weighted gene co-expression network analysis
AUC: Area under the curve
ROC: Receiver operating characteristic
